# Determinants of none-exclusive breast feeding practice among HIV positive women at selected Health Institutions in Ethiopia: case control study

**DOI:** 10.1186/s13104-019-4457-z

**Published:** 2019-07-12

**Authors:** Oumer Sada Muhammed, Kemal Ahmed Seid

**Affiliations:** 1 Department of Pharmacology and Clinical Pharmacy, School of Pharmacy, College of Health Sciences, Addis Ababa University, Addis Ababa, Ethiopia; 2 Department of Public Health, Collage of Medicine and Health Science, Wollo University, Dessie, Ethiopia

**Keywords:** None exclusive breastfeeding, HIV, Determinants

## Abstract

**Objective:**

Exclusive breast feeding (EBF) has been practiced all over the world as the best way of cost effective feeding practice, particularly in the developing countries. This practice is associated with a lower risk of human immunodeficiency virus transmission than mixed feeding. ‘Studies focusing on determinants of EBF among women living with HIV are limited. Hence, the current study is aimed at identifying those determinants.

**Result:**

This study showed that being employed (AOR = 4.363, 95% CI 2.324 to 8.191), home delivery (AOR = 0.029, 95% CI 0.004 to 0.235) and secondary education (AOR = 10.351, 95% CI 1.297 to 82.628) are significantly associated with non-EBF. In this study none EBF practice was significantly associated with women who are employed, delivered at home and educational status.

## Introduction

Breastfeeding is an unequalled way of providing ideal food for the healthy growth and development of infants; it is also an integral part of the reproductive process with important implications for the health of mothers.

EBF is defined as no other food or drink, not even water, except breast milk (including milk expressed or from a wet nurse) for 6 months of life, but allows the infant to receive ORS, drops and syrups (vitamins, minerals and medicines).

Exclusive breastfeeding for 6 months is the optimal way of feeding infants. It stimulates their immune system and protects them from diseases like diarrhea and acute respiratory infections, which the major causes of infant mortality in the developing countries [1–3].

Exclusive breast feeding (EBF) has been practiced all over the world as the best way of cost effective feeding practice, particularly in the developing countries. This practice is associated with a lower risk of Human immunodeficiency virus transmission than mixed feeding. ‘Studies focusing on determinants of EBF among women living with HIV are limited. Hence, the current study is aimed at identifying those determinants.

Despite its benefit, EBF practice is low throughout the world, where globally it is estimated that the rate of EBF is 35% [4]. Literature showed that there is an increased trend of EBF, for instance from 22% (1996) to 54% (2012) in sub-Saharan Africa, East Asia/Pacific, (excluding China) 27% (1996) to 57% (2012) and in Latin America and the Caribbean, (excluding Brazil, and Mexico) 30% to 51%, despite the reported increase of EBF, the rates are still low [1, 4–6]. The overall prevalence of EBF in Ethiopia below 6 months of age is 68% [6]. On the other hand, studies in Dares Salaam have reported EBF rates among HIV positive mothers to be 13.3% [7, 8].

Mixed feeding is associated with higher chance of HIV transmission and death rate due to diarrhea [9, 10]. Various reports showed that there is earlier initiation of complementary food in HIV exposed infants than non-exposed [11–14].

It is estimated that, EBF, 13% to 15% deaths of children under 5 years could be averted in low and middle income countries [15, 16]. Evidence showed that infants who lack EBF are more likely to be attacked and died from major causes of infant mortality like diarrhea and pneumonia. Moreover there is 3.2-fold increase risk for SAM in their childhood life time than those who got EBFWHO recommends EBF to both HIV exposed and non-exposed infants for the first 6 months of life, but still EBF rates remain low throughout the world. Globally it is estimated that prevalence of EBF is 35% [1].

A number of factors are found to affect EBF practice, these include socio-cultural factors, family and social pressures to mixed feed, customs that require giving water to newborn since every stranger entering the house is to be given water, the belief that exhaustion and thirst that the infant gets after birth necessitate giving it water and giving infants concoctions just after delivery for protection [17–19].

Accumulating evidence recommends EBF for HIV positive mother while the infant is on prophylaxis for 6 months [20, 21]. Continuation of breastfeeding up to 2 years and beyond is done in cases when child is diagnosed HIV positive whereas if the child is HIV un-infected breastfeeding is done up to 12 months and is stopped gradually ensuring that under both circumstances complementary feeding is introduced at 6th month [20].

In Ethiopia, it is a policy that all women who attend ante-natal care should be provided with free HIV counseling and testing and free provision of antiretroviral (ARV) in case if their test results are positive [22]. With these national and International efforts, the factors that lead to non-adherence to EBF among lactating women in Dessie town area are not well known. Therefore this study was aimed to assess determinants of none EBF practices among HIV positive women.

## Main text

### Methods

#### Study area and design

A facility based unmatched case control study design was employed. The study was conducted in Dessie city Administration, Amhara region, Northern Ethiopia. The administrative center of this district is Dessie city, which is located 401 km to North of Addis Ababa, the capital city of Ethiopia. Dessie city is one of the 3 metropolitan cities of Amhara region. According to 2007 Census projection It has an estimated population of 218,471, of these 49.1% are males and 50.9% are females. The study period was from February, 01, 2017 to April, 30, 2017.

#### Sample size determination and sampling procedure

The sample size was calculated using Open epidemiological information [Epi 2013 (version 7.2.0.1 CDC, Atlanta, GA) statistical software]. To calculate the sample size main independent variable antenatal care (ANC) counseling on EBF was considered for OR = 2.7. The following assumptions were also considered to calculate the sample size: 68% of controls exposed which studied in Addis Ababa 95% confidence level, 80% power of the study, and case to control ratio of 1:2, the total sample size calculated and included in the study were 249 subjects (83 cases and 166 controls) and all of them were responded 100%.

Dessie town city administration has a total of one public Hospital and eight public Health centers and several specialty and medium clinics. Out of these only three were potential (one hospital and two health centers) having large number of client flow following EPI and antiretroviral therapy (ART) services for the exposed infant clinic. So three Health facilities were fulfilling the above criteria were purposively selected in consultation with Dessie town Health office. Hence we took the study participants from each Health facilities by using population proportion sampling and the study participants were selected by convenient sampling.

- By calculating the population proportion sampling we took a total of 140 women in Dessie Hospital (46 cases and 94 control).
- We took a total of 70 women in Dessie Health center (24 cases and 46 control).
- We took also a total of 39 women in Buanbuwha Health center (13 cases and 26 control).
- Participants with non EBF were considered as cases and the controls were those with EBF.
- Participants who were not willing to participate and those with mixed feeding practice were excluded.

#### Data collection and analysis

Well-structured questionnaire was used to collect socio-demographic and economic variables for HIV positive women to assess none EBF practice for the first 6 months of infants. The questions were designed to allow mothers to express their ideas on various issues related to none EBF. The data was collected by a total of 6 nurses (2 per facility). The data quality was assured by conducting pretest and providing onsite training for data collectors.

Data were edited for accuracy, readability, consistence and completeness; thereafter it was coded and entered into a computer using software Epidata 3.1 version and then exported into Statistical Package for the Social Sciences (SPSS) version 20.0 for analysis First, Univariate analysis were done to determine various proportions including: the proportions of HIV positive mothers who practiced none EBF with various socio demographic characteristics and health related determinants. Bivariate analysis was done to measure association between the dependent variable which is none EBF and the independent variables. Variables which showed significant association of (p < 0.2) to the dependent variable (EBF) were further analyzed in multiple logistic regression model [23] to identify factors that were true association with none EBF and to control possible confounders.

#### Definition of terms

Mixed feeding: giving the baby some breast feeds, and some artificial feeds, either milk or cereal or other foods.

Pre-lacteal feeding: means of administration of any food or drinks before the first breastfeed.

HIV exposed infants: those infants born by HIV infected mothers.

Replacement feeding: infant who is not receiving any breast milk but giving nutritionally adequate diet until the age at which the child can be fully fed on family food.

#### Ethical considerations

Ethical clearance was secured from the ethical clearance committee of Wollo University, and permission from Dessie town Health office and Dessie referral hospital was obtained. The purpose, objectives, and importance of the study was explained and informed consent was secured from each participant. Confidentiality was maintained at all levels of the study.

### Results

#### Socio demographic and economic characteristics of the study participants

A total of 249 (83 cases and 166 controls) respondents were interviewed. Mean age for cases and controls was 30.9 and 30.8 years respectively. Regarding education, 5 (6%) of cases and 0 (0%) of control were not attended formal education. 7.2% of cases were cases who earned < 1000.00 birr per month whereas 2.4% was for controls. 7 (8.4%) cases came from rural area and 1 (0.6%) was for control. There were also a difference in occupational status between cases and controls that 48 (57.8%) cases were employed and 49 (25.5%) were for controls. And finally regarding on educational status of the respondents who have not formal education 5 (6%) of cases and 0 (0%) for control, similarly 28 (33.7%) cases were completed primary education but only 26 (15.7%) were controls (Table 1).

**Table 1:** Proportion and distribution of different independent variables with exclusive breastfeeding and non-exclusive breastfeeding

Variables	Frequency			
	Cases (non-exclusive BF)	%	Control (exclusive BF)	%
Age of the mother				
15–20	0	0.0	1	0.6
21–34	74	89.2	141	84.9
35–49	9	10.8	24	14.5
Residence				
Urban	76	91.6	165	99.4
Rural	7	8.4	1	0.6
Marital status				
Never married	5	6.0	6	3.6
Married	74	89.2	155	93.4
Other (divorced/widowed)	4	4.8	5	3
Educational status				
No education	5	6	0	0
Primary education	28	33.7	26	15.7
Secondary education	49	59	123	74.1
Above secondary education	1	1.2	17	10.2
Occupation				
Employed	48	57.8	49	29.5
House wife	35	42.2	117	70.5
Family monthly income in Birr				
< 1000.00	6	7.2	4	2.4
1000.00–2500.00	30	36.1	66	39.8
2501.00–5000.00	47	56.6	91	54.8
5001.00 and above	0	0	5	3
Place of delivery				
Health facility	67	80.7	165	99.4
Home	16	19.3	1	0.6
Mode of delivery				
Normal delivery	69	83.1	143	86.1
Caesarian section	7	8.4	13	7.8
Forceps delivery	7	8.4	10	6
ANC attendance during pregnancy				
Yes	74	89.2	161	97
No	9	10.8	5	3
HIV disclosure to their family				
Yes	79	95.2	165	99.4
No	4	4.8	1	0.6

*ANC* antenatal care

#### Socio demographic and economic determinants of none exclusive breastfeeding practice

On bivariate analysis, occupational status, educational status, residence were the factors found to be significantly associated with none exclusive breastfeeding practice.

In a multiple logistic regression model Employed women (AOR = 4.363, 95% CI 2.324 to 8.191), Secondary education (AOR = 10.351, 95% CI 1.297 to 82.628 and were the predictors of none exclusive breast feeding.

#### Health related characteristics of the study participants

Regarding on health related characteristics of the study participants: home delivery of 16 (19.3%) cases and 1 (0.6%) was for control, during their pregnancy period a total of 9 (10.8%) of cases were not attended ANC follow up but only 5 (3%) for controls group. 4 (4.8%) of cases were not disclosed their HIV status to the family or relatives but only 1 (0.6%) control was not disclosed their HIV status to their relatives.

#### Health related determinants of none exclusive breastfeeding

On bivariate analysis; place of delivery, ANC attendance and HIV disclosure status of the clients were found to be significantly associated with none exclusive breastfeeding practice.

In a multiple logistic regression model home delivered women (AOR = 0.029, 95% CI 0.004 to 0.235) was the predictor of none exclusive breastfeeding (Table 2).

**Table 2:** Association of women socio-demographic and other Health seeking behavior with none exclusive breastfeeding practice by bivariate and multivariate analysis in selected public facilities at Dessie town, May, 2017

Variables	Cases (n = 83)	Control (n = 166)	COR [95% CI]	AOR [95% CI]	P-value
Urban residence	76	165	15.1 [0.008–0.544]	1.2 [0.039–13.388]	0.828
Completed secondary education	49	123	6.772 [0.877–52.281]	*10.351 [1.297–82.628]*	*0.027*
Employed women	48	49	3.275 [1.892–5.668]	*4.363 [2.324–8.191]*	*0.001*
Home delivery	67	165	40 [0.003–0.195]	*0.029 [0.004–0.235]*	*0.001*
ANC attendance	74	161	3.9 [0.083–0.788]	1.635 [0.303–8.830]	0.568
HIV disclosure	79	165	8.33 [0.013–1.089]	8 [0.006–2.423]	0.169

Italic shows variables significantly associated with non exclusive breast feeding

*COR* crude odds ratio, *AOR* adjusted odds ratio, *CI* confidence interval, *ANC* antenatal care

### Discussion

The 2010 WHO infant feeding guideline has recommended that HIV-infected mothers breastfeed their infants exclusively for the first 6 months, then introduce appropriate complementary foods and continue breastfeeding for the first 12 months. In resource-limited settings, exclusive breastfeeding among HIV-infected mothers reduces infant morbidity and mortality from all causes, including HIV. Although breastfeeding by HIV infected mothers carries a risk of HIV transmission from mother-to-child, that risk decreases from 45% to less than 5% with the practice of exclusive breastfeeding and appropriate antiretroviral therapy.

This study tried to assess the association between major determinants of socio economic characteristics and other Health related factors with the practice of none exclusive breast feeding women in Dessie town.

In multiple logistic regression ANC attendance and HIV disclosure of the clients were not significantly associated with none exclusive breastfeeding. The finding of the study is similar with the report of West oromia and Addis Ababa [24, 25]. On the contrary the study conducted in Addis Ababa, Debub and Mekele [16, 25, 26] ANC attendance were significant association with none exclusive breastfeeding HIV disclosure also significant association with none exclusive breastfeeding in the study conducted in Addis Ababa and Swaziland unlike of this study [16, 27].

In this study It has been shown that educational status have influenced on the practice of EBF: women who completed secondary education found to be 10.351 times more likely to be none exclusive breastfeeding than who were no formal education. The result of this study were different with other previous study that were not associated with none exclusive breastfeeding which have done in Tanzania and Addis Ababa but similar result with studied at West showa (oromia) [18, 28]. This may be most women who are completed secondary education engaged in employment either working in government as well as private institution so as to lead few contacts with their infants for giving breastfeed exclusively.

Occupational status also has a significant association in this study that: Women who were employed were found to be 4.363 times more likely to be practiced none exclusive breastfeeding than none employed. The result of this study is similar with the one studied at Azezo and Bahirdar [29, 30] but study conducted in Addis Ababa it was not significant association with none exclusive breastfeeding. This may be due to employed women are staying most of their working hours in workplace so that the infants are exposed to other complementary foods other than breast milk.

Place of delivery is one of the predictor of EBF practice in this study that: women delivered at Home found to be 0.029 times more likely to be practice none exclusive breastfeeding than women who were delivered at the Health facilities. The result of this study was similar with other previous study which have done in Azezo, Guatemala and Bahirdar [29–31] on the other hand the study conducted in West oromia and Addis Ababa women delivered at Home was not significant association with none EBF. This result simply have shown that women delivered at the Health facilities may have an information regarding the benefit of EBF and the current guideline of infant feeding option for HIV positive women.

### Conclusion

In this study none EBF practice was significantly associated with women who are employed, delivered at Home and completed secondary education. Hence Dessie town Health office should communicate with employed organization to advocate EBF in their working place and permit adequate time for their lactating women who are employed in their organization. The town Health office also think about to promote institutional delivery in close contact with urban Health extension workers as almost all home delivered women were practicing none exclusive breastfeed.

## Limitations of the study

The major limitation of the current study is limited sample size which may affect the generalizability of the findings.

## Data Availability

The data that support the findings of this study are available from the corresponding author.

## Abbreviations

HIV: human immunodeficiency virus
ANC: antenatal care
EBF: exclusive breast feeding
