# Correction to: Determinants of none-exclusive breast feeding practice among HIV positive women at selected Health Institutions in Ethiopia: case control study

**DOI:** 10.1186/s13104-020-05074-6

**Published:** 2020-04-24

**Authors:** Aklilu Abera Ayele, Kemal Ahmed Seid, Oumer Sada Muhammed

**Affiliations:** 1 Yale Global Health Leadership Initiative, New Haven, USA; 2 Department of Public Health, College of Medicine and Health Science, Wollo University, Dessie, Ethiopia; 3 Department of Pharmacology and Clinical Pharmacy, School of Pharmacy, College of Health Sciences, Addis Ababa University, Addis Ababa, Ethiopia

## Correction to: BMC Res Notes (2019) 12:400 10.1186/s13104-019-4457-z

The authorship list on the original article [1] was incorrect and should instead show as Aklilu Abera Ayele, Kemal Ahmed Seid and Oumer Sada Muhammed. The authors apologise for this error.

The correct author contributions are shown below:
